# Low incidence of *BRAF* and *NRAS* mutations in a population with a high incidence of melanoma

**DOI:** 10.1007/s00428-023-03732-1

**Published:** 2024-01-06

**Authors:** Grace Neville, Barbara Marzario, David Shilling, Collette K Hand, Cynthia Heffron

**Affiliations:** 1 Department of Pathology, Cork University Hospital, Wilton, Cork, Ireland; 2 School of Medicine, University College Cork, Cork, Ireland; 3 Department of Pathology, University College Cork, Cork, Ireland

**Keywords:** Melanoma, *BRAF* mutation, *NRAS* mutation

## Abstract

Reported rates of *BRAF* mutation in Irish cutaneous melanoma cohorts are lower than the reported international data. We aimed to assess the mutational status of a cohort of primary cutaneous melanomas and to correlate it with clinical follow-up data.

A total of 92 cases of primary cutaneous melanoma diagnosed at a single institution in 2012 were analyzed. Regions containing common mutations in the *BRAF*, *NRAS*, *KIT*, and *KRAS* genes were investigated by PCR amplification followed by Sanger sequencing. Demographic details, tumor characteristics, and 10-year outcome data were also obtained.

Ten cases with *BRAF* V600E mutations (11.6%) and five (5.49%) *NRAS* mutations (4 at Q61R, 1 at Q61K) were detected. No statistically significant differences were noted between groups for age, gender, depth of invasion, nodal status, or recurrence status (*p* ≥ 0.05).

These findings suggest that the Irish population has a markedly lower incidence of *BRAF* and *NRAS* mutations in melanoma than those reported in other cohorts.

## Introduction

Melanoma is the fifth most commonly diagnosed cancer in Ireland, and its incidence is projected to increase significantly by 2040 [1].

Accurate identification of *BRAF* mutations has both prognostic and treatment implications [2].

Rates of *BRAF* mutation in published Irish cohorts have ranged from 19 to 29% [3–5]. This is significantly lower than reported international data which suggests a range from 40 to 60% [5–24]. The reasons for this difference are not well understood.

We aimed to assess the mutational status of a cohort of primary cutaneous melanomas diagnosed in an Irish tertiary referral center and to correlate these findings with clinical follow-up data.

## Materials and methods

With institutional review board approval; all 132 cases of primary cutaneous melanoma that were diagnosed in Cork University Hospital in the year 2012 were reviewed.

After microdissection, 92 cases progressed to DNA extraction from formalin-fixed, paraffin-embedded (FFPE) blocks using the High Pure FFPET DNA Isolation kit (Roche). Cases that did not progress included consultation cases from outside institutions (*n* = 23), those without sufficient residual tissue for further testing, or invasive melanomas so focal that microdissection was not possible.

Regions containing common mutations or variations in the *BRAF* (G469, E586, E597, V600, V601), *KRAS* (G12, G13), *KIT* (L576), and *NRAS* (G12, G13, D38, A59, Q61, K117, A146) genes were investigated by polymerase chain reaction (PCR) amplification followed by Sanger sequencing.

For validation, results were cross-referenced in cases where molecular testing for clinical purposes for *BRAF* V600 had also occurred.

Demographic details, histopathologic tumor characteristics (as recorded in synoptic summaries), and 10-year outcome data were also obtained.

Comparisons between groups including *t*-tests and Fisher’s exact tests for continuous and categorical data, respectively, were calculated using the GraphPad QuickCalcs [25].

## Results

Ninety-two cases of melanoma occurring in 91 different patients were included. Patients had a mean age of 60.9 years (± 17.5) and 48.9% were male. The most commonly seen melanoma subtype was superficial spreading (*n* = 57). The mean depth of invasion was 1.93 mm (± 3.5 mm).

Five patients had positive lymph nodes at the time of diagnosis; three were detected by sentinel lymph node sampling and two were detected clinically. One patient (with wild-type disease) had metastatic disease at diagnosis (pleural effusion positive for melanoma).

Ten other patients subsequently developed metastatic disease during the 10-year follow-up period (two of whom were positive for BRAF V600E and two of whom were positive for NRAS Q61R). One patient with wild-type disease developed liver metastases 10 years post-diagnosis; in all other cases, the metastases initially presented in lymph nodes.

Sequencing for variants in the genes of interest was successfully completed as follows: *BRAF* (*n* = 89), *KRAS* (*n* = 46), *KIT* (*n* = 44), and *NRAS* (*n* = 91), respectively. Technical and sample availability issues precluded testing of all variants in some cases (Table 1).

**Table 1:** Sequencing outcomes for each variant in genes of interest

Gene variant	WT	Mutation	ND	Sequence success (%)	Mutation (%)
*NRAS* G12	90	0	2	97.8	0.0
*NRAS* G13	90	0	2	97.8	0.0
*NRAS* D38	77	0	15	83.7	0.0
*NRAS* A59	80	0	12	87.0	0.0
*NRAS* Q61	75	5	12	87.0	6.3
*NRAS* K117	78	0	14	84.8	0.0
*NRAS* A146	75	0	17	81.5	0.0
*BRAF* G469	86	0	6	93.5	0.0
*BRAF* E586	86	0	6	93.5	0.0
*BRAF* E597	87	0	5	94.6	0.0
*BRAF* V600	76	10	6	93.5	11.6
*BRAF* V601	86	0	6	93.5	0.0
*KRAS* G12	46	0	46	50.0	0.0
*KRAS* G13	46	0	46	50.0	0.0
*KIT* L576	44	0	48	47.8	0.0

Sequence results per gene and variant investigated. Sequencing success was less than 100% which was primarily due to technical issues and DNA viability and/or availability

*WT* wild type, *ND* not determined

*BRAF* V600 testing detected 10 (11.6%) cases with a pathogenic mutation. All identified pathogenic mutations were in V600E GTG>GAG (NM_004333.6:c.1799T>A).

*NRAS* testing showed 5 cases with a mutation; 4 of which had a Q61R CAA>CGA mutation (NM_002524.5:c.182A>G), and 1 of which had a Q61K CAA>AAA (NM_002524.5:c.181C>A) mutation).

No variants were identified in cases tested for *KRAS G12* or *G13* or *KIT L576* but the sequencing success rate was only 47–50% (Table 1).

*NRAS* and *BRAF* mutations were mutually exclusive in all cases where both were successfully sequenced (*n* = 78). In all cases, a single mutation was detected.

No statistically significant differences were noted between the mutated or wild-case molecular groups for any of the mutations in terms of age, gender, depth of invasion, nodal status, or recurrence status (*p* ≥ 0.05) (Table 2).

**Table 2:** Demographic details of each of the molecular subgroups investigated

	*BRAF*		*NRAS*		*KRAS*	*KIT*
	WT (*n* = 79)	Mutant (*n* = 10)	WT (*n* = 86)	Mutant (*n* = 5)	WT (*n* = 46)	WT (*n* = 44)
Mean age (years) (range)	61.9 (20.7–95.5)	54.6 (25.2–84.2)	61.1 (20.7–95.5)	63.7 (43.7–82.6)	61.1 (20.7–89.9)	59.2 (20.7–95.5)
Gender	50.6% male	40% male	48.8% male	60% male	50% male	53.4% male
Breslow thickness (mm) (range)	1.9 (0.2–20.3)	2.4 (0.5–4.5)	1.9 (0.2–20.3)	2.8 (0.8–8.5)	2.6 (0.2–20.3)	1.8 (0.2–17)
Metastasis	15, 19%	2, 20%	16, 19%	2, 40%	11, 23.9%	5, 11.4%
Subtype *n* (%)	77 (100)	10 (100)	84 (100)	5 (100)	46 (100)	43 (100)
Superficial spreading	47 (61)	9 (90)	54 (64.3)	4 (80)	30 (65.2)	28 (65.1)
Lentigo maligna	15 (19.5)		16 (19)		6 (13)	9 (20.9)
Nodular	5 (6.5)	1 (10)	6 (7.1)	1 (20)	4 (8.7)	2 (4.7)
Acral	6 (7.8)		5 (6)		4 (8.7)	2 (4.7)
Desmoplastic	4 (5.2)		3 (3.6)		2 (4.3)	2 (4.7)
Site *n* (%)	77 (100)	10 (100)	84 (100)	5 (100)	46 (100)	43 (100)
Head and neck	22 (28.6)	2 (20)	24 (28.6)	-	15 (32.6)	12 (27.9)
Upper limb	21 (27.3)	5 (50)	23 (27.4)	3 (60)	14 (30.4)	12 (27.9)
Lower Limb	14 (18.2)	1 (10)	13 (15.5)	1 (20)	7 (15.2)	10 (23.3)
Chest and back	14 (18.2)	2 (20)	18 (21.4)	1 (20)	6 (13)	8 (18.6)
Acral	6 (7.8)	-	6 (7.1)	-	4 (8.7)	1 (2.3)
Staging *n* (%)	77 (100)	10 (100)	84 (100)	5 (100)	(*n* = 46) (100)	(*n* = 43) (100)
pT1a	33 (42.9)	2 (20)	36 (42.6)	1 (20)	15 (32.6)	16 (35.7)
pT1b	14 (18.2)	3 (30)	17 (20.2)	-	9 (19.6)	11 (26.3)
pT2	17 (22.1)	3 (30)	16 (19)	3 (60)	10 (21.7)	7 (16.7)
pT3	7 (9.1)		8 (10)		6 (13)	5 (11.9)
pT4	6 (7.8)	2 (20)	7 (8.3)	1 (25)	6 (13)	4 (9.5)

No discordant results were observed in cases where molecular testing for clinical purposes for *BRAF* V600 was performed (*n* = 13).

## Discussion

As illustrated in Table 3, the internationally quoted incidence of *BRAF* mutations in melanoma of primary cutaneous origin is 40–71%, including among other predominantly white populations [5–24]. Furthermore, some studies with reports of low incidence of *BRAF* mutations include heterogenous melanoma populations including those with mucosal melanomas which are well documented to typically have a different molecular profile [26]. We observed *BRAF* mutations in just 10 (11.2%) cases, all of which were in *V600E GTG>GAG*, confirming the previously reported markedly decreased incidence of *BRAF* mutations in cases of cutaneous melanoma in Ireland.

**Table 3:** BRAF status in comparable population cohorts

Country of the population studied	BRAF mutation (%)	Sample size	Median age (years)
Spain [6, 7]	26%, 41%	147, 264	53, 68
USA [8*, 9, 10, 11]	30%, 57%, 47%, 50%	912, 69, 677, 105	57, 58, 54, UK
Australia [12–14, 15*]	48%, 48%, 46%, 26%	197, 193,308, 1223	52, 53, 62, 60
Sweden [16, 17]	41%, 71%	203, 219	60, 61
Russia [18]	41%	80	59
Germany [19, 20]	44%, 39%	141, 437	58, 57
Italy [21]	46%	291	52
Brazil [22]	70%	77	45
Belgium [5]	43%	60	63
Ireland [5]	24%	689	Unknown
Scotland [23]	25%	52	Unknown
Turkey [24]	43%	106	Unknown
This study	11.2%	91	Unknown

*Combined US/Australian study cohort

Compared with patients with *BRAF* WT melanoma, those with *BRAF*-mutated melanoma have reportedly been more often younger and have tumors that have a thinner Breslow thickness with superficial spreading or nodular histology and/or in anatomical regions without chronic sun damage [12, 27, 28]. In keeping with these findings, cases with *BRAF* mutations in our cohort trended toward being younger, and 90% of the *BRAF*-mutated melanomas were of the superficial spreading subtype (Table 2). However, the *BRAF*-mutated melanomas in our cohort trended toward being thicker. No differences were observed in the metastatic potential of *BRAF* mutant and *BRAF* WT tumors.

The second most commonly reported *MAPK* pathway aberration in melanoma is mutated *NRAS*, occurring in ~15–30% of internationally published cases [21, 27–30].

*NRAS* mutations have previously been documented as occurring in 21% of an Irish cohort (*n* = 21) [5] which was largely in keeping with the reported incidence in other populations [5, 8–10].

Our cohort had *NRAS* mutations in just 5 (5.49%) cases (4 of which had a *Q61R* mutation and 1 of which had a *Q61K* mutation). However, the relatively few detected mutations in NRAS likely preclude certainty on the significance of these findings.

As has been previously described in patients with melanomas harboring *NRAS* mutations, the patients trended toward being older (> 55 years), had thicker primary tumors, and had more frequent metastases than tumors associated with *BRAF* mutations or wild-type tumors [21]. *NRAS* mutations are reported more frequently present in higher chronic sun-induced damage melanomas [27]. However, the *NRAS* mutated melanomas in our cohort did not appear to trend toward occurring at more sun-exposed body parts than elsewhere.

Our study has a number of limitations. The sample size is relatively small. Technical and sample availability issues precluded testing of all variants in some cases. Although coverage of the most commonly observed mutations in the genes of interest was achieved, sequencing was performed on a limited number of targeted mutations. Therefore, the possibility exists that other mutations were present and not detected. We elected to examine primary melanomas and to observe progression over 10 years. However, testing primary tumors may underestimate the mutational burden observed at the time of progression.

Data on ethnic background is not routinely recorded in our pathology databases. However, data from the 2011 census demonstrates that the Irish population at the time was 84.5% White Irish (self-identified) and 94.3% White [31]. Given that our institution serves the more rural south-west of the country; the percentage of white Irish is likely even higher—a cohort typically considered to be of “Celtic ancestry”.

A low level of *BRAF* mutations (25%) has also been found among Scottish melanoma patients [23]. Red hair, resulting from an inactivating mutation in the *MC1R* gene, has long been associated with Celtic individuals and up to 75% of the Irish population carry a variant *MC1R* mutation [32].

It has been shown that the introduction of *BRAF* V600E mutation into mice carrying the *Mc1r* mutation leads to a high incidence of invasive melanomas without providing or inducing additional gene aberrations or ultraviolet exposure [33]. Therefore, it has been hypothesized that *BRAF* mutations in the Celtic population might be reduced as a cause of genetic drift or natural selection to protect this population from melanoma [5]. However, the high incidence of BRAF-mutated melanomas has been observed in fair-skinned individuals in countries such as the USA and Australia [8–15]. A significant number of these individuals are likely to be descended from Celtic ancestors raising uncertainty as to whether genetic predisposition is the (or at least the only) factor at play here.

Effects of the changing pattern of sun exposure in the Irish population may result in differences in the molecular patterns of melanomas in this cohort in the future. With the increasing industrialization of the country and increased availability of cheap air travel, beach holidays abroad in more southern latitudes have become a common experience. This relatively elderly population from 2012 may be more reflective of melanomas that developed in the more traditional exposure pattern.

## Conclusion

These findings suggest that the Irish population has a markedly lower incidence of *BRAF* and *NRAS* mutations in melanomas than that previously reported in other population cohorts and will therefore potentially benefit less from the success of *BRAF* and *MEK* inhibitor therapy as well as any future NRAS-targeted treatments.

The findings in our study have considerable relevance to those treating melanoma in the Irish population and should be considered at a national level when developing strategies for treatment planning, budgets, and involvement in clinical trials because there will likely be more reliant on immune checkpoint inhibition as a treatment strategy in our cohort than is observed internationally.

Further investigation of the possible genetic underpinnings of our findings is warranted.

Given the large Irish diaspora internationally, these findings may have relevance in a wide number of international practice settings.
